# Recent Progress in Distributed Brillouin Sensors Based on Few-Mode Optical Fibers

**DOI:** 10.3390/s21062168

**Published:** 2021-03-19

**Authors:** Yong Hyun Kim, Kwang Yong Song

**Affiliations:** Department of Physics, Chung-Ang University, Seoul 06974, Korea; kim.yonghyun@kt.com

**Keywords:** few-mode fiber, fiber optic sensors, Brillouin scattering, distributed measurement

## Abstract

Brillouin scattering is a dominant inelastic scattering observed in optical fibers, where the energy and momentum transfer between photons and acoustic phonons takes place. Narrowband reflection (or gain and loss) spectra appear in the spontaneous (or stimulated) Brillouin scattering, and their linear dependence of the spectral shift on ambient temperature and strain variations is the operation principle of distributed Brillouin sensors, which have been developed for several decades. In few-mode optical fibers (FMF’s) where higher-order spatial modes are guided in addition to the fundamental mode, two different optical modes can be coupled by the process of stimulated Brillouin scattering (SBS), as observed in the phenomena called intermodal SBS (two photons + one acoustic phonon) and intermodal Brillouin dynamic grating (four photons + one acoustic phonon; BDG). These intermodal scattering processes show unique reflection (or gain and loss) spectra depending on the spatial mode structure of FMF, which are useful not only for the direct measurement of polarization and modal birefringence in the fiber, but also for the measurement of environmental variables like strain, temperature, and pressure affecting the birefringence. In this paper, we present a technical review on recent development of distributed Brillouin sensors on the platform of FMF’s.

## 1. Introduction

Since the first experimental demonstration of the temperature and strain measurement based on Brillouin scattering in a single-mode fiber (SMF) in late 1980s [1,2], the key performances of distributed sensors such as sensing range, spatial resolution, measurement time, and accuracy have been significantly improved over the past decades, thanks to the advances on optical methodologies, signal processing techniques, and optoelectronic devices [3,4,5,6,7]. Brillouin sensors using an SMF can provide a long measurement distance over 100 km in obtaining local Brillouin gain spectrum (BGS), however, accurate discrimination between the effects of temperature and strain variation is still challenging although a few approaches have been proposed [7,8]. In optical communications, a few-mode fiber (FMF), together with a multi-core fiber (MCF), have received considerable attention as a potential platform for space division multiplexing (SDM) [9,10], attracted by the demand for increasing transmission capacity. In optical fiber sensors as well as optical communications, different spatial modes in an FMF can find unique applications such as simultaneous measurement of multiple variables, and this has been the main target in the development of Brillouin sensors based on an FMF.

As most of commercially available fiber-optic components are made of SMF, the handling and analysis of signals in a specific higher-order mode of FMF requires an efficient and selective mode converter that couples the fundamental mode and the target higher-order mode. Several active and passive mode converters have been developed in the form of directional coupler, long-period fiber grating, and phase plate [11,12,13,14,15]. Among them a mode selective coupler (MSC) is a directional coupler that provides mode coupling between the fundamental and higher-order modes with low-loss, broad bandwidth, and high mode-purity, which is suitable for the FMF-based Brillouin sensor system [16,17]. For the methodology of distributed sensing, an optical time-domain or correlation-domain approach is used to obtain the BGS of intermodal SBS, similar to the cases of SMF-based Brillouin sensors [18,19]. The Brillouin dynamic grating (BDG), in which the acoustic phonons generated by the SBS of pump waves are used for the reflection of probe wave, can be demonstrated and characterized in various configurations using the FMF between different optical modes [20,21].

In this paper, recent experimental results of the FMF-based distributed Brillouin sensors are revisited. The spectral characteristics of the Brillouin gain spectrum (BGS) of the intra- and intermodal SBS using the LP_01_ and LP_11_ modes in the e-core TMF are presented in Section 2, where additional multi-peak features of the intermodal SBS in a circular-core FMF are also presented. Brillouin frequency measurement using optical time-domain analysis on the SBS of the e-core TMF is provided in Section 3 with the loss characterization of LP_11_ mode according to the bending radius and direction. Analysis on the double peak BGS uniquely shown in the intramodal SBS of LP_11_ mode is described in Section 4, where the optical correlation-domain analysis is applied with differential measurement schemes. The optical time-domain measurements of the intermodal BDG spectra with two LP modes of the e-core TMF are demonstrated in Section 5, where the temperature and strain coefficients of BDG frequency for different pairs of pump-probe modes are determined. The result of discriminative sensing of temperature and strain distribution is presented by applying optical time-domain reflectometry of the BDG spectrum in the e-core TMF.

## 2. Brillouin Scattering in Few-Mode Optical Fibers

Local temperature and strain variation can be measured through the spectral shift of the BGS in the distributed Brillouin sensor [22,23]. The reflection spectrum of spontaneous Brillouin scattering of the fundamental LP_01_ mode in an SMF is generally composed of multiple Lorentzian curves with the full width at half maximum (FWHM) of about 20–30 MHz [22,23,24], each of which generally corresponds to different acoustic modes in the fiber. When the configuration of Brillouin amplifier is adopted, only a single Lorentzian curve is dominantly obtained as BGS by SBS. The frequency offset between the pump and probe waves with the maximum Brillouin gain on the probe is called Brillouin frequency (*ν_B_*), which is given by [23]:

(1) $$\nu_{B}=\frac{2n_{eff}V_{a}}{\lambda }$$

where *n_eff_*, *V_a_*, and *λ* are effective refractive index (ERI), acoustic velocity, and wavelength of the light source, respectively. Since the *V_a_* of acoustic mode is a function of acoustic frequency, the *ν_B_* is theoretically determined as a common solution between optical and acoustic dispersion relations. At the wavelength of 1550 nm, ERI of 1.45, and acoustic velocity of ~5900 m/s, the *ν_B_* is calculated to be ~11 GHz. For accurate measurement of the BGS and *ν_B_* of optical fiber, one needs a narrowband (<1 MHz) light source, a photo detector (PD), microwave devices, and electro-optic modulators for the control or measurement of frequency offset between two optical waves. The localization of sensing positions in distributed Brillouin sensors additionally requires high-speed electronics like a short-pulse generator, a high-speed PD, a high-speed data acquisition system for time-domain schemes, and accurate frequency- or phase-modulation devices with a lock-in amplifier for the correlation-domain systems. Although suffered by the system complexity and high cost, Brillouin sensors can provide truly distributed measurement of strain and temperature when compared to FBG-based point sensors. The longer sensing range (over 100 km) and the higher spatial resolution (order of cm or sub-cm) are advantageous features of Brillouin sensors when compared to Raman-scattering-based distributed temperature sensor (DTS). Dependencies of *ν_B_* on temperature and strain variations are the characteristics of test fiber itself, and the coefficients of conventional SMF’s are known as ~1 MHz/°C and ~0.05 MHz/με, respectively [23]. For distributed sensing of temperature and strain by *ν_B_*, the measurement of reference values should be performed first, and at least two independent sets of measurement results with different coefficients are needed to discriminate the effects of temperature and strain. In the intermodal SBS of FMF, a higher-order spatial mode like the LP_11_ mode is used, which has anti-symmetric electric field (E-field) distribution in contrast to the symmetric distribution of the fundamental mode. Since the acoustic wave is generated by the electrostriction caused by the moving interference pattern of the E-fields of optical waves, the intermodal SBS between the LP_01_ and LP_11_ modes is necessarily intervened by anti-symmetric acoustic modes showing unique BGS with Lorentzian shape.

In a circular-core fiber, the LP_11_ mode is an approximate mode composed of almost degenerate TM_01_, TE_01_, and HE_21_ modes. This feature results in unstable orientation of the intensity lobe in the propagation along the fiber. When the core is elliptical, on the contrary, the LP_11_ mode splits into two groups, i.e., LP_11_^odd^ and LP_11_^even^ modes, with considerably different ERI’s and well-defined and stable intensity patterns. When the size and ellipticity of core are properly designed, the LP_11_^odd^ mode is cut-off while the LP_11_^even^ mode is still guided. This fiber is called e-core TMF, supporting two stable spatial modes, i.e., the LP_01_ and the LP_11_^even^ modes [25,26]. The e-core TMF is useful for several applications such as fiber sensors, tunable filters, and fiber lasers [27,28] thanks to the stable lobe orientation in the LP_11_ mode. The e-core TMF used in our work has the index difference Δ of 0.6% and the core radius of 5.4 μm × 3.6 μm, which guides only the LP_01_ and the LP_11_ even modes with the LP_11_ odd mode cut-off at 1550 nm. The polarization birefringence Δ*n* (for LP_01_ mode) is about 3.5 × 10^−5^ [16].

**Figure 1:**
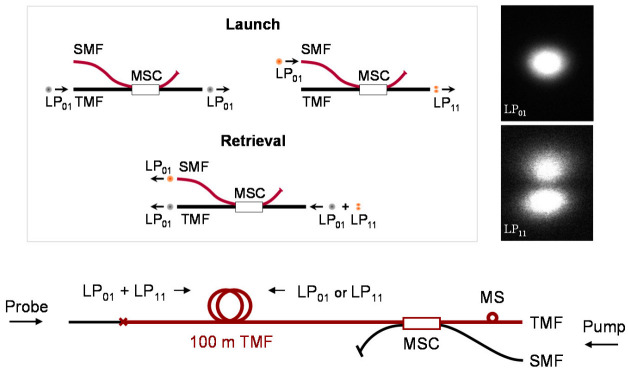
Operation of the MSC for launch and retrieval of each mode (upper), and the connection of fiber under test (FUT) and MSC for the BGS measurement of the e-core TMF. Reprinted with permission from Ref. [16] © The Optical Society.

For the measurement of BGS with the e-core TMF, a polished-type mode selective coupler (MSC) was used for the selective launch and retrieval of each mode [16]. The MSC is fabricated using a pair of SMF and e-core TMF, where the ERI of the LP_11_ mode in the e-core TMF is set equal to that of the LP_01_ mode in the SMF. Figure 1 shows the operation of MSC. The coupling efficiency between the LP_01_ mode of SMF and the LP_11_ mode of TMF was ~80%, and the purity of generated mode in the TMF was 23 dB, respectively. The inset shows the far fields from the output end of the e-core TMF measured after selective launching of each mode by the MSC, which reflects the clear images of LP_01_ and LP_11_ modes [16].

**Figure 2:**
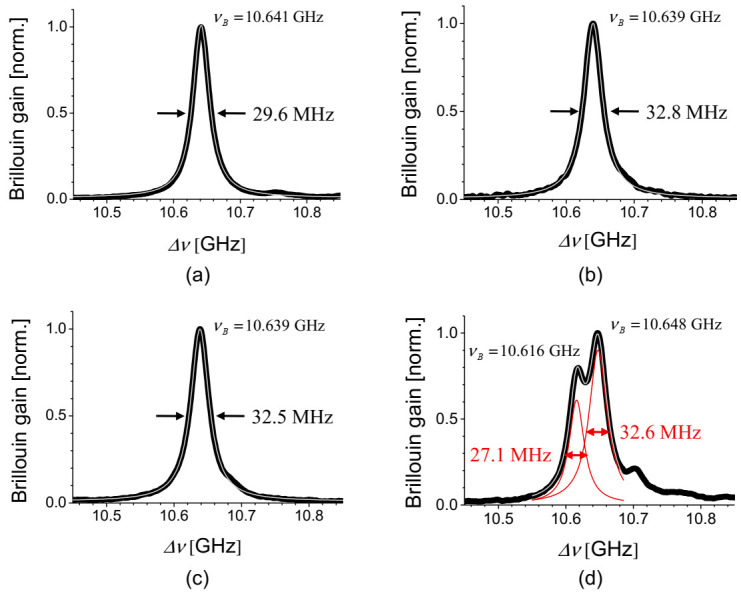
BGS of different pairs of pump-probe modes in the e-core TMF [16]: (**a**) LP_01_-LP_01_; (**b**) LP_01_-LP_11_; (**c**) LP_11_-LP_01_; and (**d**) LP_11_-LP_11_ modes. Reprinted with permission from Ref. [16] © The Optical Society.

Figure 2a–d are the BGS of four possible pairs of pump-probe modes of LP_01_-LP_01_, LP_01_-LP_11_, LP_11_-LP_01_, and LP_11_-LP_11_ modes, respectively, in the e-core TMF, showing the FWHM as indicated [16]. It is interesting to see that the BGS of the intramodal SBS of LP_01_ mode and the intermodal SBS between LP_11_ and LP_01_ modes show a single Lorentzian peak with ~30 MHz of FWHM, while the BGS of the intramodal SBS of LP_11_ mode has two dominant peaks of comparable size. Although a single peak appears in both Figure 2a,b, it should be noted that the intramodal SBS of LP_01_ mode is intervened by a symmetric acoustic mode while the intermodal SBS between LP_01_ and LP_11_ modes is by an anti-symmetric acoustic mode, due to the symmetry of E-field distribution of interacting optical modes. Such a difference can be supported by the observed ~10% difference in the FWHM, which reflects the difference in the lifetime of acoustic phonon in two scatterings. It is also thought that the multiple peaks of the intramodal SBS of LP_11_ mode are attributed to higher-order symmetric acoustic modes, and the simulation results in [16] show that the measured *ν_B_* differences in Figure 2 match well with simulations with discrepancy of less than ±3 MHz. In the FMFs, the phase-matching condition of the Brillouin scattering presented in Equation (1) is rewritten as:

(2) $$\nu_{B}=\;\left(\frac{n_{i}}{\lambda_{i}}+\frac{n_{j}}{\lambda_{j}}\right)V_{a}\approx \frac{V_{a}}{\lambda }\left(n_{i}+n_{j}\right)$$

where *n_i_*_(*j*)_ and λ*_i_*_(*j*)_ are the ERI and wavelength of *ith (jth*) mode, respectively. It should be noted that the acoustic velocity (*V_a_*) changes according to the acoustic mode.

**Figure 3:**
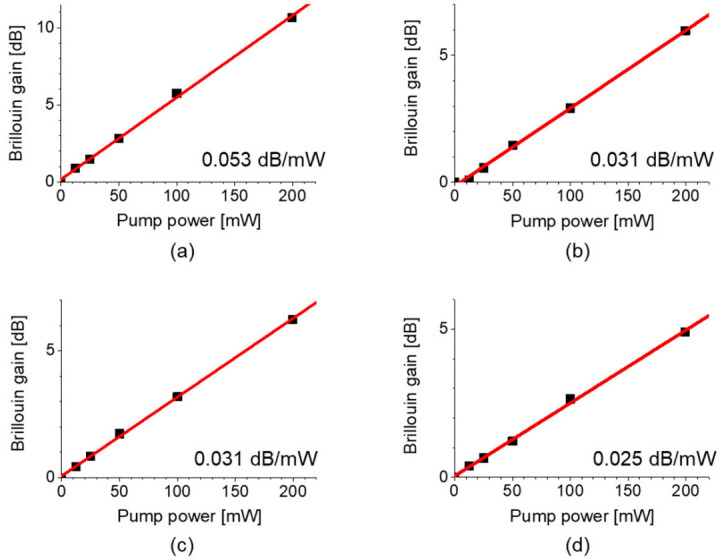
Brillouin gain as a function of pump power for the pump-probe pairs of (**a**) LP_01_-LP_01_, (**b**) LP_01_-LP_11_, (**c**) LP_11_-LP_01_, and (**d**) LP_11_-LP_11_ modes, plotted with a line fit for each. Reprinted with permission from Ref. [16] © The Optical Society.

The Brillouin gain as a function of pump power for the four different pairs is plotted in Figure 3, where the length of the e-core TMF was 100 m. When compared to the case of intramodal SBS of fundamental mode, the relative magnitude of gain of intermodal SBS (or intramodal SBS of LP_11_ mode) is 0.58 (or 0.47), both of which are large enough to be used for sensing applications [16].

**Figure 4:**
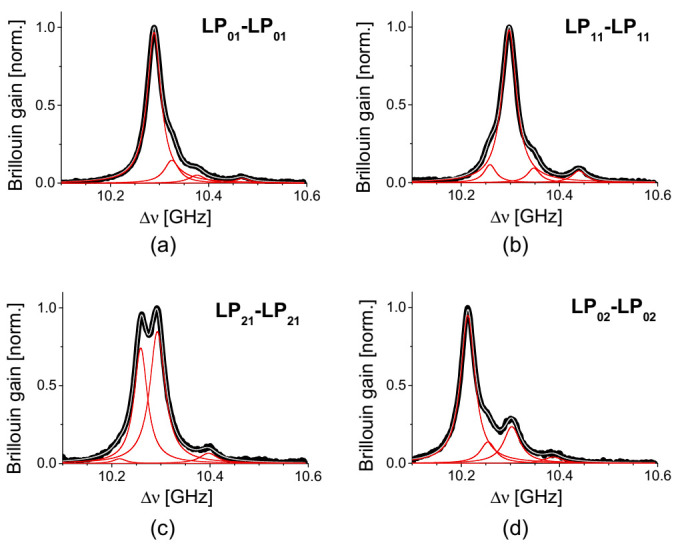
The BGS of the intramodal SBS of (**a**) LP_01_, (**b**) LP_11_, (**c**) LP_21_, and (**d**) LP_02_ modes in the FoMF. Reprinted with permission from Ref. [17] © The Optical Society.

When the fiber was changed from the e-core TMF to circular-core four-mode fiber (FoMF), more peaks were observed in the BGS, so the envelope of BGS no longer remains as a single Lorentzian in most cases of pump-probe modes [17]. Figure 4a–d are the BGS of intramodal SBS in the FoMF using the LP_01_, LP_11_, LP_21_, or LP_02_ mode for both pump and probe waves. In all four cases, four large and small peaks were found in the BGS, and in particular the BGS of LP_21_ mode shows two dominant peaks with comparable gain while the rest commonly have three small peaks with a single dominant peak. It is thought that the intramodal SBS in the FoMF is intervened by a series of symmetric acoustic modes, which is supported by the fact that the amount of frequency separation between each of the four peaks is common in all the intramodal BGS. If the order of the acoustic mode is numbered to increase from small to large frequency, those numbers for dominant acoustic mode are not identical in those four cases.

**Figure 5:**
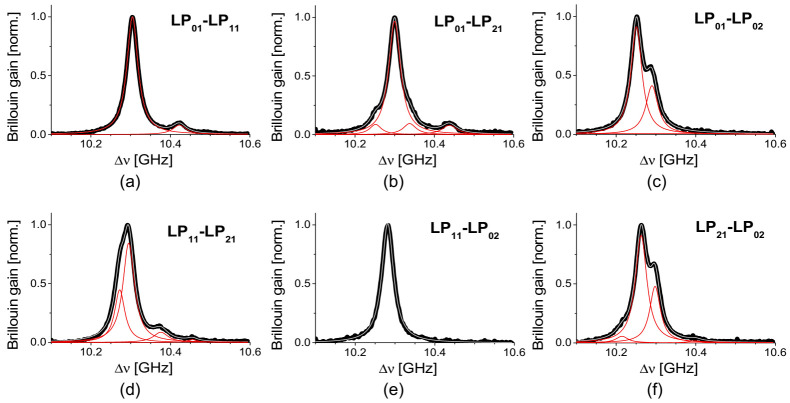
The BGS of the intermodal SBS for the pump-probe pairs of (**a**) LP_01_-LP_11_, (**b**) LP_01_-LP_21_, (**c**) LP_01_-LP_02_, (**d**) LP_11_-LP_21_, (**e**) LP_11_-LP_02_, and (**f**) LP_21_-LP_02_ modes in the FoMF, fitted with different numbers (1–3) of Lorentzian curves for each. Reprinted with permission from Ref. [17] © The Optical Society.

Multiple peaks are also observed in the BGS of intermodal SBS in the FoMF for the pump-probe pair of LP_01_-LP_11_, LP_01_-LP_21_, LP_01_-LP_02_, LP_11_-LP_21_, LP_11_-LP_02_, and LP_21_-LP_02_ as presented in Figure 5a–f [17]. Unlike the intramodal SBS, the BGS of the intermodal SBS can be fitted with different numbers (from 1 to 4) of Lorentzian curves.

The relative magnitude of the gain coefficients of the intramodal (or intermodal) SBS in the FoMF were reported within 35–55% (or 14–45%) of that of the LP_01_ mode [17]. To build a distributed Brillouin sensor system using a FoMF, the pump-probe mode pair should be carefully selected to secure a clear and large signal free from possible intermodal interference. The difference of the SBS threshold from that of the fundamental mode in the FoMF was measured to be 0.6, 2.7, and 2.8 dB for the LP_11_, LP_21_, and LP_02_ mode, respectively [17].

Additionally, the measurement of the BGS of a circular-core two-mode fiber was reported, applying free-space mode coupling [29], and the results show single Lorentzian gain curves for both intra- and intermodal SBS. It is thought that further investigation is needed for the quantitative analysis on the acoustic modes involved in the SBS of FMF’s.

## 3. Optical Time-Domain Brillouin Sensor Using a Few-Mode Fiber

Optical time-domain reflectometry and analysis have been the most popular way for implementing distributed Brillouin sensors, thanks to the simple configuration and intuitive signal structure compared to frequency and correlation domain methods [1,2,3,30]. The time of flight of the pump pulse directly corresponds to the sensing position, and the spatial resolution of the system is determined only by the duration of pulse. In long-distance applications of FMF’s, the propagation loss of a higher-order mode is expected to be significantly larger than that of the fundamental mode, and its distribution, as well as the distribution of other ambient variables, is measurable for each mode by applying Brillouin optical time domain analysis (BOTDA).

### 3.1. Bending Loss Characteristic of the LP_11_ Mode in the E-Core TMF

**Figure 6:**
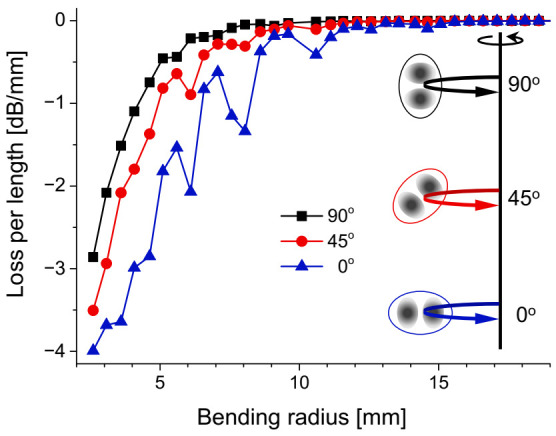
Loss of the LP_11_ mode per unit length in the e-core TMF according to bending radius [32].

The optical loss induced by an unwanted macro-bending could become a potential weakness either in communication or a sensor based on higher-order spatial modes. Some periodic notches are commonly detected in transmitted optical power as the bending radius is reduced, which is known as the resonances of the whispering gallery modes at the core/cladding boundary [31]. The bending loss characteristics of the LP_11_ modes in the e-core TMF were measured with polyethylene mandrel and rotation stages. Figure 6 shows the measurement result according to the bending radius with three different bending directions relative to the orientation of core ellipse when half-round bending is applied [32].

The mandrel was made as a co-axial cylinder having a diameter varying from 5.2–46.2 mm with 1 mm steps. Three different angles of 0°, 45°, and 90° between the plane of the bending and major axis of the elliptic core were applied to check the loss characteristics. When the plane of bending is parallel to the major axis of core (i.e., 0°), one can observe some periodic notches were observed in the transmitted power with overall the largest of loss among three cases. Meanwhile, the notches disappear when the plane of bending becomes orthogonal (i.e., 90°) with overall the smallest loss. Such orientation-dependent loss is thought to originate from the π-phase difference between the E-fields of two intensity lobes, which may suppress the coupling to whispering gallery modes by the offset of two contributions at 90°.

### 3.2. Distributed Brillouin Sensor for Bending Loss Detection

**Figure 7:**
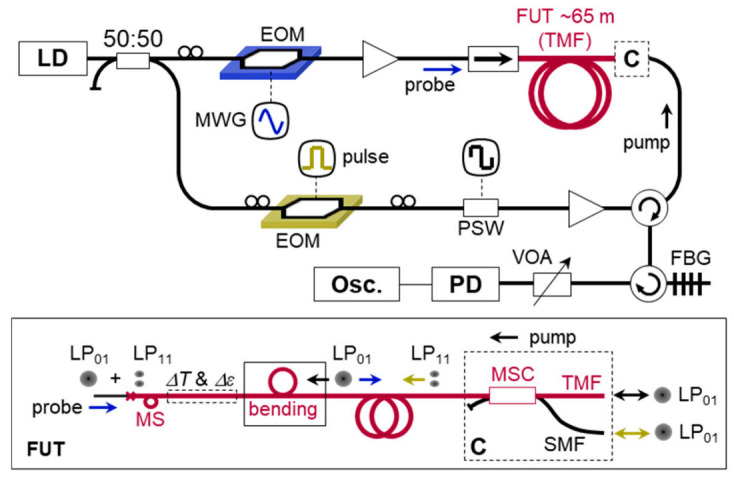
Setup of the BOTDA system based on the e-core TMF [32]: EOM, electro-optical modulator; MWG, microwave generator; PSW, polarization switch; FBG, fiber Bragg grating; VOA, variable optical attenuator; PD, photo detector; FUT, fiber under test; MSC, mode selective coupler.

Brillouin optical time-domain analysis (BOTDA) and reflectometry (BOTDR) are simple and intuitive ways for distributed Brillouin sensing. The experimental setup of the BOTDA system based on the e-core TMF is presented in Figure 7 [32]. A distributed feedback laser diode (DFB-LD) with a center wavelength of 1550 nm was used as a light source, and the output was divided by into the pump and probe arms. The probe was generated by an electro-optic modulator (EOM) and a microwave generator (MWG), and the pump was modulated as a pulse with a duration of 20 ns corresponding to the spatial resolution of 2 m. The polarization of the pump was switched between two orthogonal states of polarization by a polarization switch (PSW) for acquiring the average value of Brillouin gain. The LP_01_ mode was launched as the probe to the fiber under test, a 65 m e-core TMF, through a mode stripper (MS), i.e., tight bend, and the LP_01_ and LP_11_ modes were selectively launched as the pump from the opposite end by using an MSC as depicted in the inset ‘FUT’. The MSC was also used to selectively retrieve the LP_01_ and LP_11_ mode components of the probe for signal acquisition. A fiber Bragg grating (FBG) was used to filter out the anti-Stokes components of two sidebands.

**Figure 8:**
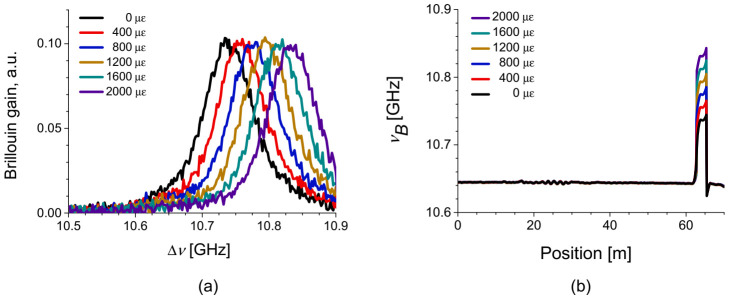
(**a**) BGS at the test section with different strains for the intermodal SBS. (**b**) Distribution map of *ν_B_* with variable strains at the test section for the intermodal SBS [32].

The variation of *ν_B_* with respect to temperature and strain change was measured from the single-peak BGS of the intra- and intermodal SBS, as seen in Figure 2. Examples of the measured BGS at the strain-applied section and the distribution map of *ν_B_* along the fiber are plotted in Figure 8a,b, respectively, where variable strain with 400 με step was applied to the 2.5 m test section near the end of FUT. The average *ν_B_* of the intermodal SBS in the FUT was ~10.642 GHz [32].

**Figure 9:**
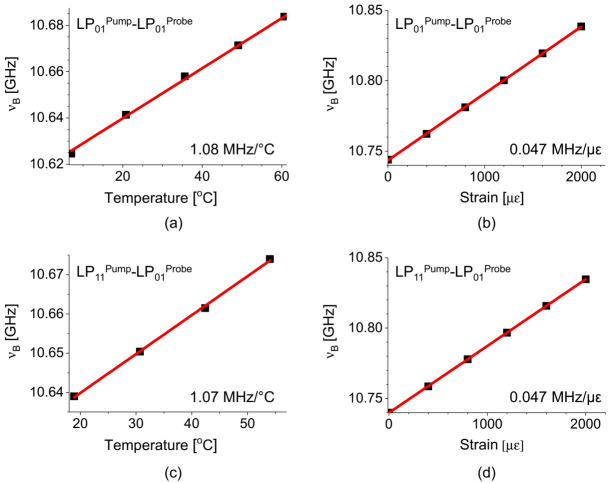
Temperature and strain dependence of the *ν_B_* for (**a**,**b**) the intramodal SBS of LP_01_ mode and (**c**,**d**) the intermodal SBS between LP_11_ and LP_01_ modes, respectively, in the e-core TMF [32].

The temperature (C*_T_*) and strain (C*_ε_*) coefficients of the *ν_B_* for different pairs of pump-probe modes were measured by the experimental setup in Figure 7, and the results are presented in Figure 9a,b for the pump-probe pair of LP_01_-LP_01_, and Figure 9c,d for the LP_11_-LP_01_, respectively [32]. The measured coefficients of 0.047 MHz/με for strain and 1.07 (or 1.08) MHz/°C for temperature are similar to those of conventional SMF, and the distinction between the intra- and intermodal SBS is negligible, which indicates that it would not be possible to discriminate the temperature and strain by using those two pairs of the e-core TMF.

**Figure 10:**
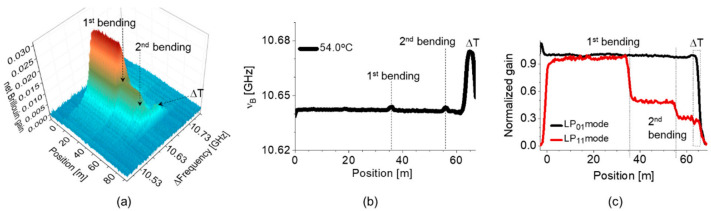
Simultaneous measurement of the loss of LP_11_ mode and *ν_B_* based on the e-core TMF: (**a**) the distribution map of BGS of intermodal SBS, (**b**) the distribution map of *ν_B_* of intermodal SBS, and (**c**) the distribution map of Brillouin gain normalized by the maximum for the intra- (black, LP_01_ mode) and intermodal (red) SBS [32].

Although the diameter of unwanted macro-bending cannot be quantified due to the existence of resonance notches, the presence of the bending loss is detectable by measuring the gain reduction in the intermodal SBS. Two different bends with diameters of 1.4 and 1.5 cm were applied at the position of 36 and 56 m from the input port of the pulse for simultaneous measurement of the loss of LP_11_ mode and *ν_B_*. The measurement results are plotted in Figure 10, where Figure 10a,b show the distribution maps of the BGS and *ν_B_* of the intermodal SBS, and Figure 10c is the map of Brillouin gain normalized to its maximum for the intra- (black, LP_01_ mode) and intermodal (red) SBS, respectively. One can see the change of signal by two local bends, such as the small variation of *ν_B_* in Figure 10b and the abrupt reduction of the Brillouin gain in Figure 10a,c. The shift of *ν_B_* near the rear end of FUT is observed in Figure 10a,b by intentional temperature change from 25 to 54 °C [32].

**Figure 11:**
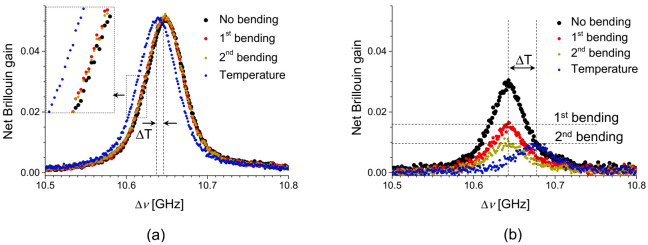
Local BGS at the different positions after bending of zero, one, and two turns and temperature variation after the second bending for (**a**) the intramodal (LP_01_ mode) and (**b**) the intermodal SBS [32].

Local BGS measured at different positions are shown in Figure 11a,b, respectively. Figure 11a is the BGS of intramodal (LP_01_ mode) SBS where the BGS in black, red, and green correspond to the positions of 35, 38, and 58 m after zero, one, and two turns of bending, respectively, at ambient temperature of 25.5 °C. The BGS in blue is the result at 65 m with the temperature of 18.2 °C, also after two turns of bending. One can see the shift of BGS while the Brillouin gains are maintained. Figure 11b is the BGS of intermodal SBS where the BGS in black, red, green, and blue represent the results of the same positions as those in Figure 11a except that the temperature was 30.8 °C for the result at 65 m (blue). In intermodal SBS, the gain reduction is clearly observed, which is measured to be 49% and 39%, respectively, by the first and second bending at different diameters (14.1 and 15.1 mm). It is notable that the peak gain of intermodal SBS (black) was about 59% of that of the SBS between LP_01_ modes, matching well with the result obtained by CW interaction of the pump and probe waves [16].

It is worth mentioning that the ratio of Brillouin gain of the intermodal SBS to that of the intramodal (LP_01_ mode) SBS is 0.6 in a circular core TMF [29], which is quite similar to 0.58, the ratio of the e-core TMF [16]. Additionally, the strain coefficients of intra- (LP_01_ mode) and intermodal SBS (LP_01_ and LP_11_) of the e-core TMF were almost the same as 470 MHz/%, while quite dissimilar values were reported for the intramodal SBS (592 MHz/% for LP_01_, and 487 MHz/% for LP_11_ mode, respectively) of a circular-core FMF [15]. The BOTDA based on the circular-core FMF has been successfully applied for the discriminative measurement of strain and temperature, thanks to the large discrepancy in the strain coefficients [15]. It is notable that the measurement with a circular-core FMF shows better accuracy in discriminating the effects of two environmental variables than that with the e-core TMF. However, the difficulties in the development of optical components and the low stability of Brillouin interaction for the LP_11_ mode in the circular-core fiber, due to the unstable lobe orientation, are other important issues to be solved for its practical applications. The BOTDA system based on the FMF provides an advantage of simultaneous measurement of bending loss, strain and temperature variations, while the TMF-based OTDR system can measure only the loss distribution of the higher order mode [33].

## 4. Optical Correlation-Domain Brillouin Sensor Using a Few-Mode Fiber

There are three different pairs of pump-probe modes for the intra- and intermodal BGS in the e-core TMF, and the temperature and strain dependences of *ν_B_* distribution for two pairs (LP_01_-LP_01_ and LP_11_-LP_01_) were measured applying the BOTDA system. It is necessary to change the sensing scheme to investigate the temperature and strain dependence of the double-peak BGS of the intramodal SBS of LP_11_ mode in the e-core TMF, presented in Figure 2d, since the frequency separation (~32 MHz) between two peaks is too close [32].

### 4.1. Brillouin Optical Correlation-Domain Analysis with Differential Measurement Scheme

**Figure 12:**
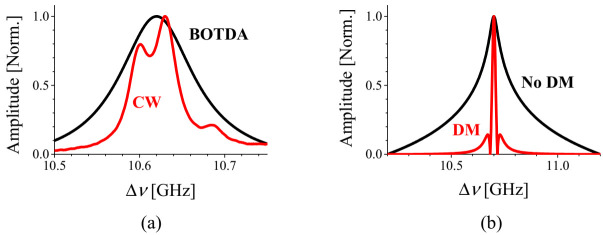
(**a**) Double-peak BGS of the LP_11_ mode measured by CW pump and probe waves (red) and by a BOTDA system with a 20 ns pulse as the pump (black), respectively. (**b**) Example of BGS of an SMF measured by a BOCDA using the ordinary configuration (black) and the DM with a 5 MHz phase modulation (red) [32].

The FWHM of BGS is reported to be about 30 MHz at the wavelength of 1550 nm in a conventional SMF. In general, the BGS measured by an optical time-domain Brillouin sensor is the spectral convolution of the intrinsic Lorentzian BGS and the spectrum of pump wave. The spectral width of BGS measured by a BOTDA system is generally broader than the intrinsic spectral width (i.e., ~30 MHz) due to the finite spectral width of the pump pulse [34]. Figure 12a shows the BGS of the intramodal SBS of the LP_11_ mode, with a continuous wave (CW) pump (red) and a 20 ns-pulsed pump (black) [32]. It is seen that the double peak structure in the CW-based BGS completely disappears when the pulsed pump is applied. Therefore, one needs another approach to characterize the double-peak BGS of intramodal SBS of the LP_11_ mode.

The Brillouin optical correlation-domain analysis (BOCDA) has been developed for high-resolution distributed Brillouin sensing with CW pump and probe waves on the basis of synthesis of optical coherence function (SOCF) for localizing the sensing position [19]. Differential measurement (DM) is a modified lock-in detection technique that was introduced to the BOCDA system for improving the spatial resolution and dynamic range of distributed measurement [35]. The DM-BOCDA system is operated by applying on-off phase modulation to the pump to suppress the noise substructure in the BGS. An additional notable advantage of the DM-BOCDA is the acquisition of BGS with a spectral width narrower than the intrinsic Brillouin linewidth of 30 MHz. Figure 12b shows examples of BGS obtained by the ordinary (black) and the DM-based (red) BOCDA systems for an SMF, where the FWHM of signal obtained by the DM-BOCDA system is only about 17 MHz while that of ordinary BOCDA system is as large as 170 MHz.

### 4.2. BOCDA System Based on the Intramodal SBS of LP_11_ Mode

**Figure 13:**
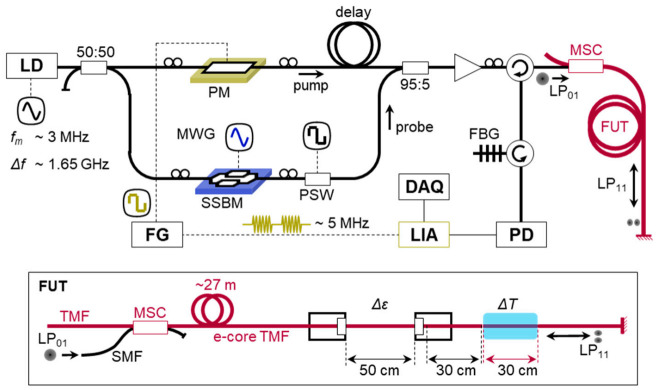
Experimental setup of the BOCDA using the e-core TMF [32]: PM, phase modulator; FG, function generator; SSBM, single sideband modulator; MWG, microwave generator; PSW, polarization switch; MSC, mode selective coupler; FUT, fiber under teste; FBG, fiber Bragg grating; PD, photo detector; LIA, lock-in amplifier; DAQ, data acquisition.

Figure 13 is the experimental setup of the DM-BOCDA based on the intramodal SBS of LP_11_ mode in the e-core TMF [32], which is a linearly configured system using a 1550 nm DFB-LD as the light source. The modulation frequency (*f_m_*) and depth (Δ*f*) were about 3 MHz and 1.65 GHz, respectively, which corresponds to the measurement range of 33 m and the nominal spatial resolution of 20 cm [19].

The output from DFB-LD was divided by a 3 dB coupler for the probe and pump waves, which were separately modulated by using a single sideband modulator (SSBM) and a phase modulator (PM), respectively. In particular, the frequency of the phase modulation of pump was set as 5 MHz that was periodically turned on and off at 91 kHz, and this on-off frequency was used as the reference frequency of a lock-in amplifier (LIA) for DM. The frequency offset of the probe was swept from 10.5 to 10.8 GHz for the acquisition of BGS. The pump and probe waves were combined and launched to an FUT (e-core TMF) from one end in the LP_11_ mode through an MSC. The intramodal SBS of the LP_11_ mode occurred between the outgoing pump and the incoming probe (i.e., 4% Fresnel reflection from the cleaved end of FUT). The probe component was selected by an FBG among the reflected waves, and it was received by a PD for lock-in detection.

**Figure 14:**
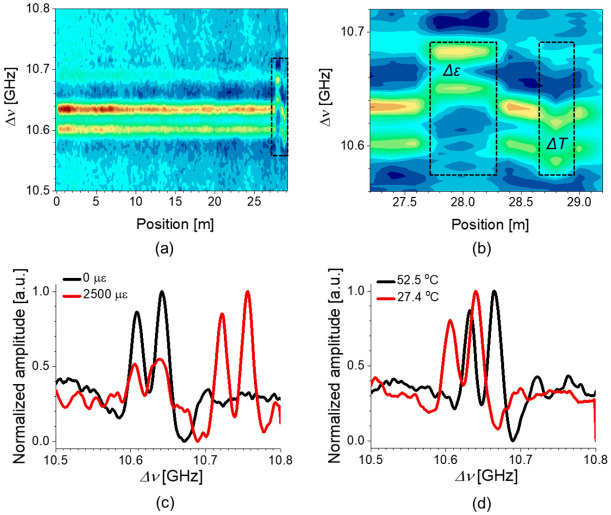
(**a**) Distribution map of the double-peak BGS measured by the DM-BOCDA system. (**b**) Zoomed view of the 2-m test section near the end of FUT. Local BGS of the intramodal SBS of LP_11_ mode at the position where (**c**) strain and (**d**) temperature variations were applied [32].

The distribution of the BGS of the intramodal SBS of LP_11_ mode measured by the DM-BOCDA system is plotted in Figure 14a, and the zoomed view of the test sections (dashed box) near the end of FUT is shown in Figure 14b. One can observe the shift of double-peak BGS according to the variations of temperature (Δ*T* = 12.9 °C) and strain (Δ*ε* = 1000 με) applied to the spans of 30 cm and 50 cm, respectively. Figure 14c,d show the examples of local BGS at the test section, where the double-peak structure and the shift of the BGS of intramodal SBS of LP_11_ mode are clearly seen with different strains (0 and 2500 με) and temperatures (27.4 and 52.5 °C) applied.

**Figure 15:**
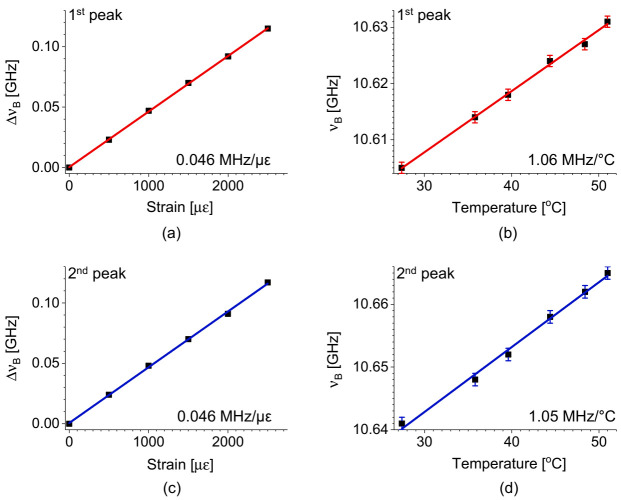
The shift of *ν_B_* of the double-peak BGS in the intramodal SBS of LP_11_ mode, for the first peak according to (**a**) strain and (**b**) temperature, and for the second peak according to (**c**) strain and (**d**) temperature, respectively [32].

Figure 15a–d show the strain and temperature dependence of *ν_B_* for the intramodal SBS of LP_11_ mode, measured separately for each peak in the double-peak BGS, where we set the peak order according to the *ν*_B_’s from low to high. The strain and temperature coefficients of the first (second) peak were measured as 0.047 MHz/με (for both), and 1.06 (1.05) MHz/°C, respectively. It is notable that the measured coefficients of the two peaks are very close to those of the intermodal and intramodal SBS of fundamental modes with the discrepancy less than 1%. Since the two peaks in the intramodal SBS of LP_11_ mode have nearly the same coefficients for ambient variables, it is expected that the intramodal SBS of LP_11_ mode can be also used for the BOTDA system, by ignoring the double-peak structure and applying single-peak fitting to the combined BGS.

The BOCDA using FMF might be applicable to distributed sensing with a cm-order spatial resolution for discriminative measurement, if the relative amplitude or frequency separation of two peaks in the intramodal BGS of LP_11_ mode shows a significantly large discrepancy in the coefficients of two different physical variables. Another possible application is the development of linearly configured BOCDA system applying intermodal SBS, where selective reflection of each optical mode at the end of FUT could be used as the counter-propagating wave.

## 5. Brillouin Dynamic Grating Sensor Based on a Few-Mode Fiber

Brillouin dynamic grating (BDG) represents an acoustic phonon, which is generated in the process of SBS of optical waves called ‘pump’ and plays the role of a moving grating for another wave called ‘probe’ [20]. Several applications have been reported on the basis of BDG such as tunable delay lines, microwave filters, all-optical signal processing, and distributed sensors [36,37,38,39,40,41,42,43,44]. The BDG is typically implemented using a birefringent medium like a polarization maintaining fiber (PMF), and in the reflection spectrum of BDG (or BDG spectrum) the frequency offset between the pump and probe waves is called BDG frequency (*ν_D_*), which is a function of birefringence. The sensors based on BDG measure the ambient variables like strain, temperature, and pressure through the change of local birefringence [39,40,41,42,43,44]. When compared to ordinary Brillouin sensors, the BDG-based sensors can provide about 20 and 50 times better sensitivities for the strain and temperature measurement with conventional PMF’s [39,40], and distributed sensors of hydrostatic pressure have also been reported as applying the BDG, where the sensitivity is at least 60 times higher than that of ordinary Brillouin sensors [42]. In 2012, the operation of BDG applying different spatial modes of an FMF has been demonstrated [21], where the number of possible combinations of the pump-probe pairs is increased according to the number of optical modes guided in the FMF. In this intermodal BDG operation, the grating is written by one spatial mode and used to reflect another spatial mode at an optical frequency different from the pump.

**Figure 16:**
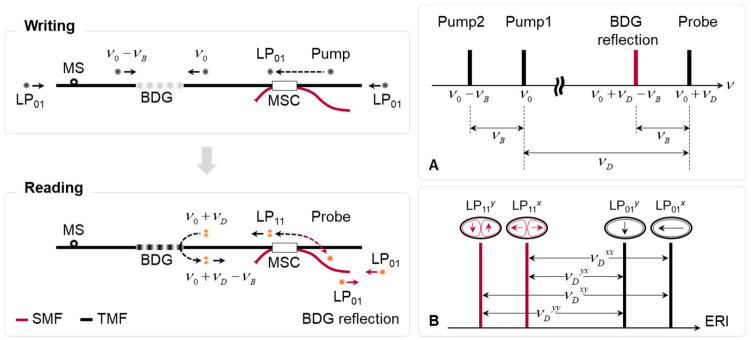
Schematic of the intermodal BDG operation in the e-core TMF. Inset A: Spectral relation of pump and probe waves. Inset B: Four possible pairs of pump-probe modes in the e-core TMF resulting in four different BDG frequencies [43].

Figure 16 shows the schematics of the intermodal BDG operation in the e-core TMF, where the writing (upper) and reading (lower) procedures of the acoustic grating using the LP_01_ and LP_11_ mode are described, respectively [43]. The MSC was used to launch the pump in the LP_01_ mode for writing the BDG, and to launch and retrieve the probe in the LP_11_ mode to read out the grating reflection. The insets A and B denote the spectral relation of four optical components and the four different BDG frequencies occurring in the operation of BDG based on the e-core TMF, respectively.

The phase-matching condition of the intermodal BDG operation is that the Brillouin frequency of the pump in the LP_01_ mode is equal to that of the probe in the LP_11_ mode. One can rewrite Equation (2) for the pump and probe as follows [43]:

(3) $$\begin{array}{l} \begin{array}{cc} \mathrm{Pump}: & \nu_{B}=\frac{V_{a}}{\Lambda }=\frac{V_{a}}{c}\left(n_{01}\left(\nu_{0}\right)\cdot \nu_{0}+n_{01}\left(\nu_{0}-\nu_{B}\right)\cdot \left(\nu_{0}-\nu_{B}\right)\right) \end{array}\\ \begin{array}{cc} \mathrm{Probe}: & \nu_{B}=\frac{V_{a}}{\Lambda }=\frac{V_{a}}{c}\left(n_{11}\left(\nu_{0}+\nu_{D}\right)\cdot \left(\nu_{0}+\nu_{D}\right)+n_{11}\left(\nu_{0}+\nu_{D}-\nu_{B}\right)\cdot \left(\nu_{0}+\nu_{D}-\nu_{B}\right)\right) \end{array} \end{array}$$

where *n*_01_ (*n*_11_), Λ, *c*, and *ν*_0_ are the ERI of LP_01_ (LP_11_) mode, acoustic wavelength, speed of light, and the optical frequency of pump1, respectively. After Taylor expansion and some rearrangement, one obtains a simplified expression for *ν_D_* in this intermodal BDG as follows [43]:

(4) $$\nu_{D}=\frac{n_{01}\left(\nu_{0}\right)-n_{11}\left(\nu_{0}\right)}{n_{g11}}\cdot \nu_{0}\equiv \frac{\Delta n}{n_{g11}}\cdot \nu_{0}$$

where *n*_g11_ is the group index of LP_11_ mode at the optical frequency of *ν*_0_.

While the *ν_D_* is determined only by the polarization birefringence in a PMF, the *ν_D_* in a FMF is determined by both intermodal and polarization birefringence. The e-core TMF is also a kind of PMF due to the geometry of core, where two eigenstates of polarization exist for each spatial mode. Therefore, four different pairs of the pump-probe modes (LP_01_^x^-LP_11_^x^, LP_01_^x^-LP_11_^y^, LP_01_^y^-LP_11_^x^, and LP_01_^y^-LP_11_^y^) are possible for the intermodal BDG operation in the e-core TMF. As an example, when Δ*n* between two spatial modes of 3.6 × 10^−3^ and *n*_g11_ of 1.45 are used at the wavelength of 1550 nm, the *ν_D_* in the intermodal BDG is calculated to be as high as 480 GHz.

### 5.1. Optical Time-Domain Analysis of BDG Spectrum Based on FMF

**Figure 17:**
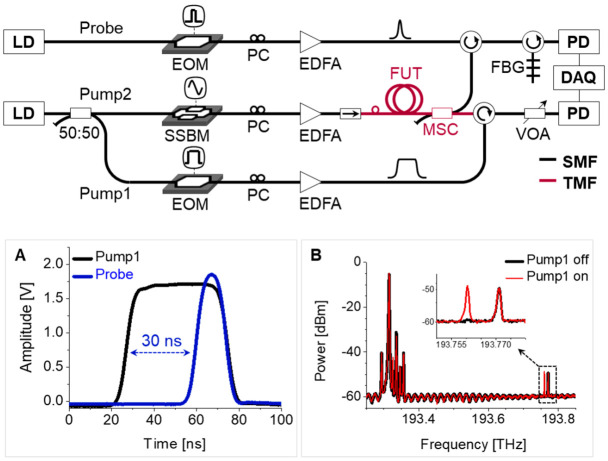
Experimental setup for optical time-domain analysis of intermodal BDG spectrum [43]. Inset A: Timing of the pump1 and probe pulses. Inset B: BDG spectrum measured at the position of the FBG. Reprinted with permission from Ref. [43] © The Optical Society.

The experimental setup for the optical time-domain analysis of intermodal BDG is depicted in Figure 17 [43], in which two DFB-LD’s with center wavelengths of 1550 and 1547 nm, were used for the pump and probe wave, respectively. The output from the pump was applied to build a BOTDA configuration using an SSBM and EOM, similar to the setup presented in Figure 7, where the only difference is the change of the modulator used to sweep the frequency offset from EOM to SSBM. The duration and peak power of the pump and probe pulse were 50 and 15 ns, and 24 and 27 dBm, respectively. The power of the CW pump2 was around 15 dBm. The state of polarization of the pump1, pump2, and probe was separately controlled by a polarization controller (PC) before being launched to the FUT (i.e., e-core TMF) through an MSC. The frequency offset between the pump and probe was swept by the current control of the pump LD with a step of 4 MHz for measuring BDG spectra. The inset A shows the timing of pump and probe pulses at the end of FUT, which was controlled to maximize the signal amplitude, and the inset B is the optical spectrum measured by using an optical spectrum analyzer (OSA) at the position of the FBG where the BDG reflection is seen in the dashed box.

**Figure 18:**
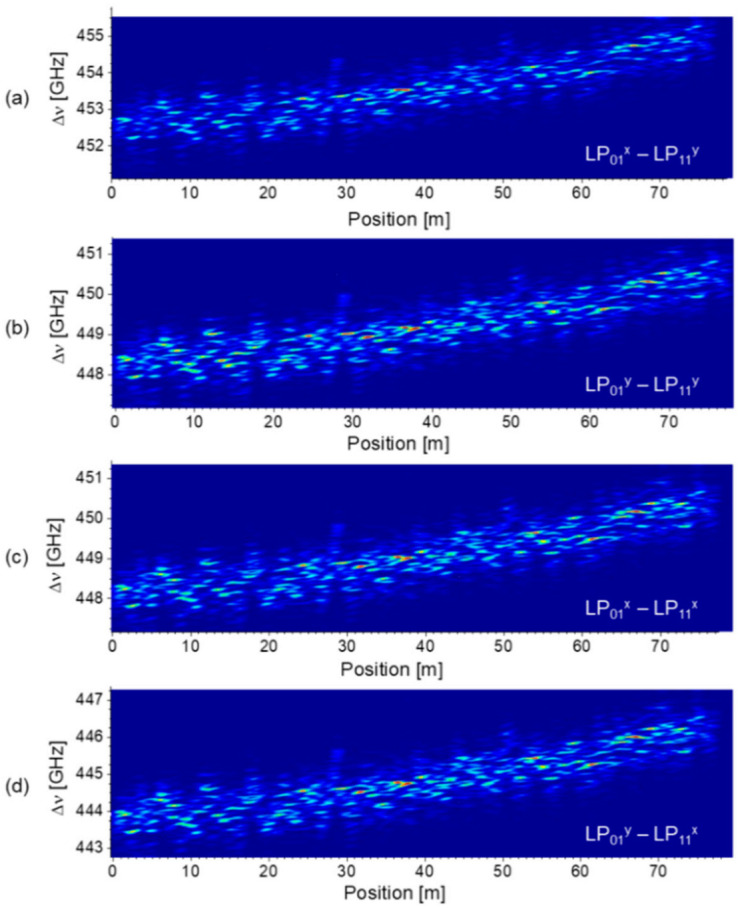
Distribution maps of the intermodal BDG spectra of the e-core TMF measured by the BDG-OTDA system, for the pump-probe pairs of (**a**) LP_01_^x^-LP_11_^y^, (**b**) LP_01_^y^-LP_11_^y^, (**c**) LP_01_^x^-LP_11_^x^, and (**d**) LP_01_^y^-LP_11_^x^ modes. Reprinted with permission from Ref. [43] © The Optical Society.

As described in inset B of Figure 16, the *ν_D_*’s in the intermodal BDG operation can be classified into three groups, the highest, two middles, and the lowest, corresponding to the pump-probe pairs of LP_01_^x^-LP_11_^y^, LP_01_^x^-LP_11_^x^, LP_01_^y^-LP_11_^y^, and LP_01_^y^-LP_11_^x^ modes, respectively. The polarization control of each optical wave is crucial, and one can also identify the state of polarization by monitoring the variation of reflection power according to the polarization control at specific frequency offset between the pump and probe waves. Figure 18a–d show the distribution maps of the BDG spectra for the pump-probe pairs of LP_01_^x^-LP_11_^x^, LP_01_^x^-LP_11_^y^, LP_01_^y^-LP_11_^x^, and LP_01_^y^-LP_11_^y^ modes, respectively, measured by the BDG-OTDA system [43]. The local BDG spectrum commonly has multiple peaks within a frequency span of ~2 GHz, which is thought to originate from the non-uniformity of the FUT with the spatial resolution (2 m) of the system. Gradual increase in the center frequency of local spectra along the FUT is observed in all four cases, which is thought to reflect the gradual change of the geometric parameters of e-core TMF in the preform fabrication or drawing process.

**Figure 19:**
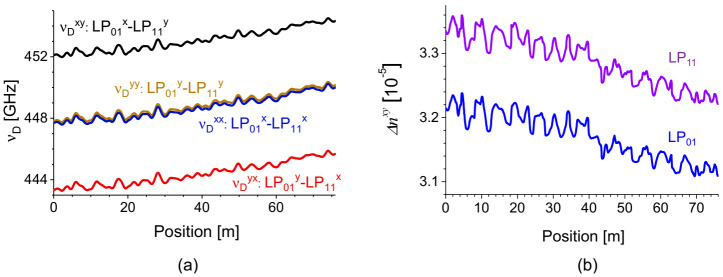
(**a**) Distribution map of *ν_D_* for different pairs of pump-probe modes of intermodal BDG. (**b**) Distribution map of polarization birefringence of two LP modes reconstructed from (a). Reprinted with permission from Ref. [43] © The Optical Society.

The distribution maps of *ν_D_* obtained by applying the center of mass fitting to the local BDG spectra in Figure 18 are plotted in Figure 19a, which show the variations of the intermodal birefringence along the fiber between the pump and probe modes. The distribution of polarization birefringence for each LP mode can also be acquired from the maps of *ν_D_* by the following formulas [43]:

(5) $$\begin{array}{c} \nu_{D}{}^{xy}-\nu_{D}{}^{xx}=\frac{\left(n_{01}{}^{x}-n_{11}{}^{y}\right)-\left(n_{01}{}^{x}-n_{11}{}^{x}\right)}{n_{g11}}\cdot \nu_{0}\equiv \frac{\Delta n_{11}}{n_{g11}}\cdot \nu_{0}\\ \nu_{D}{}^{xy}-\nu_{D}{}^{yy}=\frac{\left(n_{01}{}^{x}-n_{11}{}^{y}\right)-\left(n_{01}{}^{y}-n_{11}{}^{y}\right)}{n_{g11}}\cdot \nu_{0}\equiv \frac{\Delta n_{01}}{n_{g11}}\cdot \nu_{0} \end{array}$$

The distribution map of polarization birefringence calculated from the results of Figure 19a is plotted in Figure 19b for each LP mode, where gradual decrease of birefringence along the fiber is commonly observed as well as 1–2% local fluctuations. The polarization birefringence of LP_11_ mode is ~4% larger than that of the LP_01_ mode, while both have similar local fluctuations. Considering that the *ν_D_* of conventional PMF’s such as PANDA and bow-tie fibers, is around 45 GHz (Δ*n* of ~3.5 × 10^−4^) [45], one can see that the intermodal birefringence of the e-core TMF in Figure 19a is almost 10 times larger than the polarization birefringence of PMF’s, and the contribution of the polarization birefringence in Figure 19b is less than 1% of the overall intermodal birefringence in the e-core TMF.

**Figure 20:**
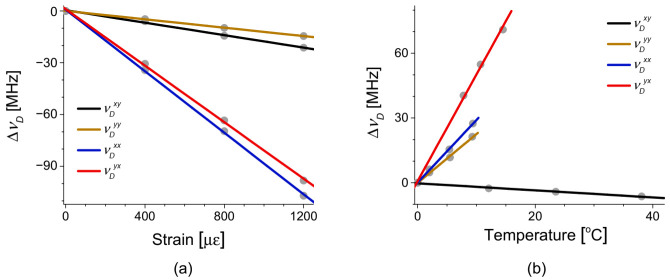
(**a**) Strain- and (**b**) Temperature-dependence of *ν_D_* of intermodal BDG for different pairs of pump-probe modes in the e-core TMF. Reprinted with permission from Ref. [43] © The Optical Society.

Figure 20a,b show the strain and temperature dependence of *ν_D_* for different pairs of pump-probe modes, measured using two test sections near the rear end of the FUT with lengths of 2 m and 5 m for strain and temperature, respectively. The coefficients for the pump-probe pairs of LP_01_^x^-LP_11_^y^, LP_01_^y^-LP_11_^y^, LP_01_^x^-LP_11_^x^, and LP_01_^y^-LP_11_^x^ modes were −0.018, −0.012, −0.089, and −0.081 MHz/με for strain and −0.16, 2.3, 2.9, and 4.9 MHz/°C for temperature, respectively. It is worth mentioning that one of the temperature coefficients is negative with the others positive, while all of the strain coefficients are negative. For references, the strain and temperature coefficients of conventional PMF’s are all positive and all negative, respectively [39,40,45]. Additionally, the coefficients are overall much smaller than those of the PMF’s, and the origin of which needs further investigation, although it is thought to be somewhat related to the smaller polarization birefringence of the e-core TMF.

### 5.2. Optical Time-Domain Reflectometry of the BDG in a Few-Mode Fiber

The discrimination of temperature and strain variations based on BDG has been experimentally demonstrated by simultaneously measuring both *ν_D_* and *ν_B_* using a conventional PMF [40,41,44]. One of possible drawbacks in the distributed BDG sensor based on PMF is the requirement of two optical systems each to acquire the information on the *ν_D_* and *ν_B_* distribution, respectively. On the other hand, the FMF-based BDG sensor has a potential to discriminate the temperature and strain by measuring multiple *ν_D_*’s, for which only a single optical system is required, so it could provide a cost-effective solution. In addition, the analysis-type sensing systems such as BOTDA and BDG-OTDA commonly have a loop configuration, although some exceptions have been reported [46], so they require launching of optical waves to an FUT from both ends. As an alternative approach, the optical time domain reflectometry of BDG spectrum (BDG-OTDR) has been proposed in 2012, which not only allows the single end access to the FUT but also simplifies the experimental setup by removing an extra modulator [47].

**Figure 21:**
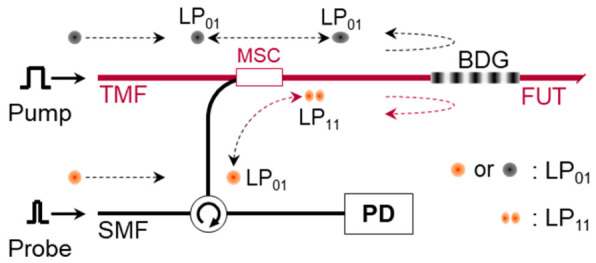
Schematic of the BDG-OTDR system based on the e-core TMF [48].

Figure 21 is the schematic of the BDG-OTDR system based on the e-core TMF [48]. The BDG-OTDR does not use any microwave device since the BDG is generated by the amplified spontaneous Brillouin scattering (ASBS) of the pump pulse, while the power and duration of pump pulse should be larger to secure enough signal amplitude for detection, compared to the BDG-OTDA. Similar to the case of BDG-OTDA, the LP_01_ and LP_11_ modes were used as the pump and probe waves, each of which was selectively launched and retrieved by the MSC.

One can decide the best pair of pump-probe modes for intermodal BDG to perform a discriminative measurement of temperature and strain considering the condition number of transfer matrix [49], and the pump-probe pairs of LP_01_^x^-LP_11_^x^ and LP_01_^y^-LP_11_^x^ modes in the e-core TMF were applied for the demonstration of discriminative sensing in [48].

**Figure 22:**
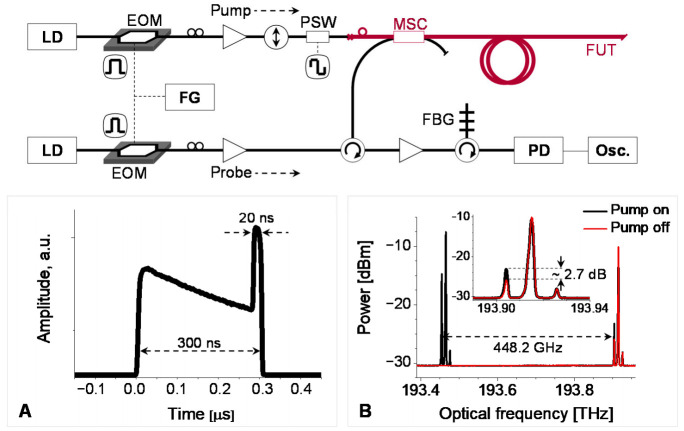
Experimental setup of the BDG-OTDR based on the e-core TMF. Inset A: Timing and duration of the pump (300 ns) and probe (20 ns) pulses. Inset B: Optical spectrum measured at the position of FBG. Reprinted with permission from Ref. [48] © The Optical Society.

The experimental setup of BDG-OTDR is presented in Figure 22. Two different DFB-LD’s, with a center wavelength of 1549 and 1546 nm, were used as light sources for the pump and probe, respectively. The pump and probe waves were modulated as a pulse with a duration of 300 ns and 20 ns, respectively, by EOM’s, and the pump and probe pulses were amplified by EDFA’s to the peak level of over 30 dBm. As shown in inset A, the propagations of two pulses were synchronized to maximize the signal amplitude. The optical spectrum measured at the position of FBG is shown in the inset B, where, as observed in the zoomed view, the Stokes wave of the spontaneous Brillouin scattering of probe is about 2.7 dB amplified by the intermodal BDG reflection while it is still 13 dB smaller than the Rayleigh scattering of probe. The average value of *ν_D_* was about 448.2 GHz.

**Figure 23:**
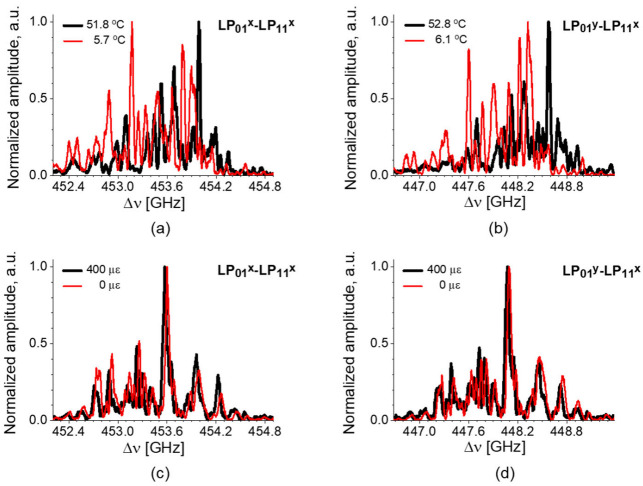
The shift of local BDG spectrum by temperature variations for the pump-probe pairs of (**a**) LP_01_^x^-LP_11_^x^ and (**b**) LP_01_^y^-LP_11_^x^ modes, and by strain variations for (**c**) LP_01_^x^-LP_11_^x^ and (**d**) LP_01_^y^-LP_11_^x^ modes. Reprinted with permission from Ref. [48] © The Optical Society.

The local BDG spectra and their shift by the strain and temperature variations are depicted as a function of frequency offset (Δν) between the pump and probe in Figure 23. The BDG spectra of the LP_01_^x^-LP_11_^x^ and LP_01_^y^-LP_11_^x^ modes are shown in Figure 23a,b with temperature variations, and Figure 23c,d with strain variations, respectively [48]. It is seen that both BDG spectra move to the lower frequency when positive strain is applied in Figure 23c,d, while the spectral shift is not clear under the temperature variations in Figure 23a,b due to the arbitrary change of the relative amplitude of side peaks.

**Figure 24:**
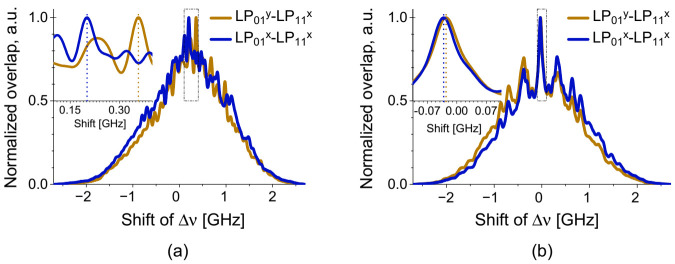
Examples of the cross correlation fitting applied to the multi-peak BDG spectra to quantify the change of *ν_D_* under the variations of (**a**) temperature and (**b**) strain.

The cross-correlation fitting is an effective way to quantify the amount of shift for the multi-peak BDG spectra [50]. Figure 24a,b are examples of the cross correlation fitting applied to the experimental results presented in Figure 23a–d, respectively. The single dominant peak in Figure 24b indicates the clear shift in spectral domain under the strain variation, and it is also feasible to apply in the case of temperature variation, through the existence of the peak in Figure 24a.

Accurate discrimination of strain and temperature variations has been a challenging task from the early stage of researches on distributed Brillouin sensors, and several techniques have been proposed applying simultaneous measurement of two independent properties [40,41,44,51]. The BDG-OTDR based on FMF also has been reported as a potential tool for the discriminative measurement, by applying temperature (*C_T_*) and strain (*C_ε_*) coefficients of different pairs of pump-probe modes [48]. The pump-probe pairs of LP_01_^x^-LP_11_^x^ and LP_01_^y^-LP_11_^x^ modes in the e-core TMF have shown different values of *C_T_*, *C_ε_* of + 4.3 MHz/°C, −0.093 MHz/με and + 7.6 MHz/°C, and −0.085 MHz/με, respectively. For the discriminative measurement of strain and temperature variations, the inverse matrix is calculated using four coefficients as follows:

(6) $$\left(\begin{array}{c} \Delta \varepsilon \\ \Delta T \end{array}\right)=\frac{1}{\det\left|\begin{array}{cc} C_{\varepsilon }{}^{xx} & C_{T}{}^{xx}\\ C_{\varepsilon }{}^{yx} & C_{T}{}^{yx} \end{array}\right|}\left(\begin{array}{cc} C_{T}{}^{yx} & -C_{T}{}^{xx}\\ -C_{\varepsilon }{}^{yx} & C_{\varepsilon }{}^{xx} \end{array}\right)\left(\begin{array}{c} \nu_{D}{}^{xx}\\ \nu_{D}{}^{yx} \end{array}\right)=\left(\begin{array}{cc} I_{\varepsilon }{}^{xx} & I_{\varepsilon }{}^{yx}\\ I_{T}{}^{xx} & I_{T}{}^{yx} \end{array}\right)\left(\begin{array}{c} \nu_{D}{}^{xx}\\ \nu_{D}{}^{yx} \end{array}\right)$$

The accuracy of the discriminative sensing can be evaluated by the condition number [49], and it is calculated as ~50 for the BDG-OTDR system, which is similar to that of the scheme using *ν_D_* and *ν_B_* with PMF [44].

**Figure 25:**
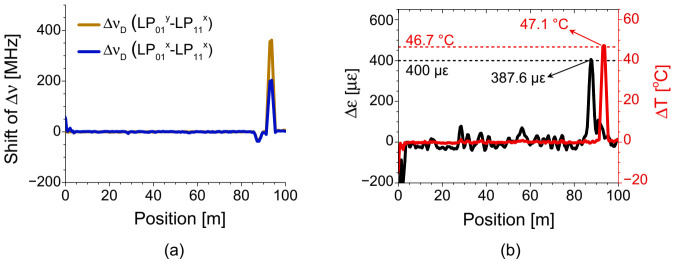
(**a**) Distribution map of the Δ*ν_D_* under temperature and strain variations applied to the test sections near the end of FUT for different pairs of pump-probe modes. (**b**) The distribution map of temperature and strain variations reconstructed from the result of (**a**) by Equation (6). Reprinted with permission from Ref. [48] © The Optical Society.

Figure 25 shows the experimental results of discriminative sensing by the BDG-OTDR, where Figure 25a is the distribution map of two Δ*ν_D_*’s under the strain and temperature variation applied at test sections near the end of a 95 m FUT [48]. The distribution map of strain and temperature variations reconstructed by the matrix calculation for each position is shown in Figure 25b, with dotted lines indicating the strain and temperature variations of 400 με and 46.7 °C applied to the test sections. The calculated strain and temperature at those positions were 386.7 με and 47.1 °C, respectively, and the discrepancies of 13.3 με and 0.4 °C were less than the error range of ±105 με and ±1.6 °C originating from the frequency drift between two light sources.

The presented method has been a unique way so far to obtain the distribution maps of both intermodal and polarization birefringence simultaneously, which, as we believe, could be useful in designing, manufacturing, and evaluating circular-core or polarization-maintaining FMF’s and related products.

## 6. Conclusions

Recent advances on the development of distributed Brillouin sensors based on few-mode fibers (FMF’s) have been reviewed, where the experimental results on the characterization of intra- and intermodal Brillouin scatterings, Brillouin sensor systems, the characterization of intermodal Brillouin dynamic grating (BDG), and BDG-based sensor systems have been presented on the basis of the elliptical core two-mode fiber. We think the FMF is a highly flexible platform for distributed sensing, which can support various combinations of optical modes for intermodal SBS and BDG, and the use of a high-performance mode coupling device like the mode selective coupler in this work is crucial for implementing the FMF-based Brillouin or BDG sensor system.

Further research needs to be done on the design parameters of the FMF optimized for Brillouin sensors that can provide strain, temperature, and pressure coefficients sufficiently different for various intra- and intermodal SBS, and is applicable to accurate discrimination of the effects of ambient variables. The axial uniformity of the waveguide properties of e-core FMF could be a significant issue in long-range sensing applications. Also, the requirement of using specialty fiber like the e-core TMF and FMF could possibly deteriorate the practicality of FMF-based distributed sensors in terms of availability and cost. We think a commercially available FMF, such as the PANDA-type PMF at a wavelength of 2 μm, might be useful as the PANDA-FMF at the communication wavelength, providing an alternative to the e-core FMF, with possibly better axial uniformity.

## Data Availability

The data presented in this study are available on request from the corresponding author. The data are not publicly available as they involve the subsequent application of patent for invention and the publication of project deliverables.
